# “Unpacking” cultural differences in social anxiety between Japanese and European Americans: the roles of threat appraisal and attentional bias

**DOI:** 10.3389/fpsyg.2023.1132918

**Published:** 2023-09-05

**Authors:** Alexander Krieg, Yiyuan Xu

**Affiliations:** ^1^Department of Global Communication, Kobe Gakuin University, Kobe, Japan; ^2^Department of Psychology, University of Hawaii at Manoa, Honolulu, HI, United States

**Keywords:** social anxiety, culture, threat appraisal, attentional bias, Japanese

## Abstract

**Introduction:**

Cultural differences in self-reported social anxiety between people of East Asian heritage and European heritage may be related to differences in independent and interdependent self-construals, which potentially influence the processing of social threat.

**Methods:**

We examined the roles of two different aspects of threat bias: threat appraisal (Study 1) and attentional bias (Study 2) to explain cultural group differences in social anxiety between Japanese and European American college students.

**Results:**

Study 1 demonstrated that sequential mediations of lower independent self-construal and higher appraisal of threat among Japanese could explain their higher social anxiety compared to European Americans. However, Study 2 failed to find the relation between cultural group differences in self-construals and attentional bias. In addition, the cultural group differences in attentional bias were unexpectedly due to stronger selective attention toward neutral stimuli among European Americans, rather than bias toward social threat among Japanese. After selective attention was experimentally manipulated, there were significant cultural group differences in self-reported social anxiety and anxious behavior in a speech task.

**Discussion:**

These conflicting findings suggested that an alternative theoretical framework other than the self-construal theory might be needed to fully account for cultural differences in attentional bias in explaining cultural group differences in social anxiety.

## Introduction

Abundant research has demonstrated that people of East Asian cultural heritage report higher social anxiety symptoms relative to people of European cultural heritage (for meta-analyzes see Krieg and Xu, 2015; Woody et al., 2015). To date, the most influential theoretical framework that has been used to explain these cultural group differences is based on the cultural model of the self in relation to others (Markus and Kitayama, 1991); That is, on average individuals of East Asian heritage (AH) are more likely to endorse interdependent and less likely to endorse independent self-construal than individuals of European heritage (EH), which may lead to more experiences of social anxiety among AH than EH (Okazaki, 1997). More recent research has begun to “unpack” the mechanism underlying the relations between cultural differences in self-construals and social anxiety, with a particular emphasis on the role of threat bias (Dalgleish, 1994; Krieg and Xu, 2018). Threat bias refers to differential attentional allocation toward and/or selective processing of threatening stimuli in a given situation (Dalgleish, 1994; Williams et al., 1997; Mathews and Mackintosh, 1998; Mogg et al., 2000; Cisler and Koster, 2010). There is preliminary evidence to suggest that cultural differences in self-construals may be related to more biases in processing social threat among AH, which in turn explain why they report higher social anxiety than EH (Krieg and Xu, 2018).

Despite the theoretical appeal of this hypothesis, there are at least two important remaining issues. First, threat bias may be manifested in different ways, such as biased appraisal (e.g., overestimating the likelihood of occurrence or negative sequences of a social situation) or biased attention (e.g., preferentially attending to social threat) (Dalgleish, 1994; Mogg et al., 2000). It is unclear which aspects of threat bias AH and EH may vary in the degree to which they report social anxiety symptoms. Second, to our knowledge, examination of cultural differences in threat bias and social anxiety had mostly relied on non-experimental evidence. It would help elucidate the role of threat bias if it can be experimentally manipulated to show its impact on cultural differences in social anxiety. Therefore, the purposes of the current studies were to investigate the relations among cultural group differences in self-construals, threat bias, and social anxiety in Japanese and European Americans.

### Cultural group differences in social anxiety between individuals of east Asian (AH) and European heritage (EH)

Social anxiety refers to the experience of intense fear and avoidance related to evaluation by others during social interactions or performances (Good and Kleinman, 1985; Mesquita and Frijda, 1992; American Psychiatric Association, 2013). The extant research has shown fairly consistent endorsements of higher social anxiety symptoms among AH relative to EH (for meta-analyzes see Krieg and Xu, 2015; Woody et al., 2015). In one meta-analysis, the Cohen’s *d* effect size ranged from *d* = 0.05 to 0.53, with an average effect size of *d* = 0.36, 95% confidence interval (CI) [0.27, 0.44] (Krieg and Xu, 2015). Furthermore, Krieg et al. (2018) demonstrated that this cultural difference in social anxiety was not an artifact of nonequivalent measurement properties between the two groups. Using a series of measurement invariance analyzes (Little, 1997; Vandenberg and Lance, 2000), Krieg (2018) were able to replicate higher social anxiety among AH than EH by comparing latent group means.

### Explaining cultural group differences in social anxiety: the self-construal theory

The self-construal theory (Markus and Kitayama, 1991) posits that in general Western societies tend to socialize values such as autonomy, uniqueness, and personal rights that contribute to a sense of self that is relatively independent from others (i.e., *independent self-construal*; Markus and Kitayama, 1991; Singelis, 1994). Contrasting independent self-construal is *interdependent self-construal*, where it is posited that traditional East Asian cultural values focus on social harmony and heightened sensitivity toward other’s feelings, opinions, and evaluations during social encounters (Fung, 1999). Although many studies have found cultural group differences in these two types of self-construals between AH and EH (e.g., Okazaki, 1997; Norasakkunkit and Kalick, 2002; Hong and Woody, 2007; Ho and Lau, 2011), there are notable exceptions, such as a study by Norasakkunkit et al. (2012), which found statistically significant group differences in independent self-construal but not interdependent self-construal. Likewise, self-construal theory has attracted some strong critiques from other researchers in the field as having weak empirical support even among studies that show group differences (Matsumoto, 1999).

As individuals view themselves as more interdependent and less independent, they place increased value on the social relationships with other people, and consequently may have elevated fears about disrupting this relationship or being negatively evaluated by others (Okazaki, 1997; Mak et al., 2011). Therefore, based on the self-construal theory, it was hypothesized that higher interdependent and lower independent self-construal may result in AH to experience more symptoms of social anxiety than their EH counterparts (Okazaki, 1997). For instance, Ho and Lau (2011) examined the relations between self-construals and social anxiety among EH and two AH groups (foreign- and U.S-born Asian Americans), and showed that interdependent self-construal was positively related to social anxiety whereas independent self-construal was negatively associated with social anxiety. It should be noted, however, Ho and Lau (2011) focused on the relations of *individual differences* in self-construals to *individual differences* in social anxiety, as moderated by AH-EH group membership, rather than directly testing how cultural *group differences* in self-construals may help explain AH-EH *group differences* in social anxiety.

Given the unexplained cultural group differences in social anxiety, the promising candidacy of self-construal theory in explaining this difference, as well as the inconsistencies in the extant research, a direct examination of the mediating influence of independent and interdependent self-construals on cultural groups differences is warranted.

### Self-construals and threat bias

As fundamental ways of thinking about the self in relation to others, self-construals may help form an interpretative framework within which social cues, particularly ambiguous and threatening ones, are attended to and appraised, and thus provide a way to understand cultural *group differences* in various aspects of threat bias such as selective attention and threat appraisal. Although few studies have directly compared AH and EH in threat bias, there is increasing evidence to suggest that to a large extent processes of selective attention to and appraisal of emotionally salient stimuli are shaped by culturally-tuned socialization experiences that reflect the predominant cultural models of self-construals. For instance, the AH-EH differences in interdependent and independent self-construals are in line with the evidence on the cultural *group differences* in selective attention to background objects or general context, with AH on average being more likely to orient their attention to the context than EH (Masuda and Nisbett, 2001; Chua et al., 2005), and are consistent with the findings that AH and EH differ at the *group* level in their behavioral and neurological responses to emotional faces indicative of social threat (Huang et al., 2001; Chiao et al., 2008).

The etiological theories of social anxiety, at the same time, emphasize processes of threat bias such as threat appraisal and attentional bias in explaining *individual differences* in social anxiety (Dalgleish, 1994). Mogg et al. (2000) proposed that it is of both theoretical and practical importance to distinguish attentional and cognitive processes in response to emotionally salient stimuli such as social threat. The bottom-up processes involve mostly selective or biased attention toward social threat, whereas the top-down process involves mostly cognitive appraisal and evaluation of social threat. Research has demonstrated that both processes of selective attention and threat appraisal precipitate and maintain the anxiety process (Wong and Rapee, 2016; also see Bar-Haim et al. (2007) for a meta-analysis).

To integrate both the cultural *group differences* in self-construals and selective attention, and *individual differences* in threat bias, Krieg and Xu (2018) proposed and tested a mediation model that connects cultural group differences in self-construals to social anxiety via the mediating role of one aspect of threat bias, threat appraisal (i.e., cultural group differences in self-construals → cultural group differences in threat appraisal → cultural group differences in social anxiety). In their study of 310 Asian American and 249 European American undergraduate students, Krieg and Xu (2018) showed that higher interdependent self-construals and lower independent self-construals among Asian Americans were related to their higher self-reported threat appraisal, which in turn partially explained (mediated) their higher self-reported social anxiety symptoms than European Americans. These findings suggest that the manner in which members of different cultural groups tend to view themselves among others influences both the degree to which situations are perceived as threatening as well as the degree to which social anxiety is experienced. However, Krieg and Xu (2018) only focused on self-reported appraisal of social threat but did not examine attentional bias toward social threat.

### Attentional bias toward social threat

While self-reports of threat appraisal could help us understand cultural group differences in cognitive and conscious evaluation of social threat, it does not capture the process of attentional bias to social threat that seems to be more effortless and sometimes unconscious (Dalgleish, 1994; Mogg et al., 2000). Attentional bias refers to social threat being prioritized in attention over other stimuli for further processing and is typically measured by attentional probe tasks such as the dot-probe discrimination task (MacLeod et al., 1986). This task consists of presenting pairs of stimuli briefly and then measuring reaction times to a subsequent target stimulus (see Study 2 Methods for further details). This experimental paradigm is commonly used in studies examining the role of attention in anxiety processes. A meta-analysis showed that attentional biases were much more prominent among anxious individuals in comparison to non-anxious individuals (Bar-Haim et al., 2007). In addition, manipulating attention toward or away from threat in laboratories or clinical settings, resulted in increased or decreased social anxiety (Amir et al., 2008; Amir and Bomyea, 2010; Heeren et al., 2011, 2013).

While few studies have directly compared AH and EH in attentional bias, it seems plausible that higher interdependent and lower independent self-construals among AH may increase their tendency to direct attention toward social threat that may interfere with their relationships with others (Chiao et al., 2008); such heightened attentional bias could lead to more experiences of social anxiety symptoms among AH than EH.

Assuming that stronger attentional bias toward social threat among AH represents an important underlying mechanism explaining cultural group differences in social anxiety between AH and EH, experimental procedures such as Attention Bias Modification Training (ABMT; MacLeod et al., 2002; Amir et al., 2009; Amir and Conley, 2014), which involves random assignment and repeatedly redirecting participants’ attention away from or toward socially relevant threat cues, may be particularly instrumental in revealing the role attentional bias may play in explaining cultural group differences in social anxiety. Research has shown that experimentally reduced or induced attentional bias via ABMT could function to influence not only self-reported subjective experiences of social anxiety, but also anxious behavior and physiological arousal in response to social stressors (e.g., MacLeod et al., 2002; Amir et al., 2008, 2009; Heeren et al., 2012a). Although these studies had been largely limited to differentiating high and low anxiety within one cultural group, this paradigm could be useful to understand cultural group differences between groups that report higher vs. lower levels of social anxiety. Additionally, ABMT has been similarly utilized in research with AH participants (e.g., Japanese; Irie et al., 2015), suggesting at least a base level of cultural validity.

### The Japanese cultural context

Japan is characterized by high population density and insular social groups that are relatively difficult to join or leave (Yuki and Schug, 2012). Consistent with an interdependent model of self-construal, unlike most Western cultures there is an overall emphasis on norm following, hierarchical relationships, and protecting group harmony (Gelfand et al., 2011), which may influence the way how social threat is attended to and appraised in the Japanese culture. In addition, past research has demonstrated relatively higher social anxiety symptoms reported by Japanese than European Americans (e.g., Norasakkunkit and Kalick, 2009), making the Japanese culture an ideal context to further explore AH-EH group differences in social anxiety in relation to self-construals and threat bias.

### The current studies

We conducted two studies to understand the role of threat bias, as manifested in threat appraisal (Study 1) and attentional bias (Study 2), in explaining cultural group differences in social anxiety between Japanese and European Americans. As a replication of Krieg and Xu (2018), Study 1 explored the role of threat appraisal in the relation between cultural group differences in self-construals and social anxiety using situation sampling and self-reports. Study 2 examined whether Japanese tended to exhibit stronger attentional bias toward threat than European American, and if so, how experimentally training attention to orient away from or toward social threat (Amir et al., 2008, 2009; Koster et al., 2009; Heeren et al., 2012b; Woud and Becker, 2014) may differentially impact cultural group differences in not only self-reported social anxiety but also behavioral and physiological markers of social anxiety.

## Study 1

Study 1 focused on appraisal of social threat in social anxiety-evoking situations. We expected (1) Japanese to report higher interdependent but lower independent self-construal, higher threat appraisal, and higher social anxiety symptoms than European Americans; and (2) the relations between cultural group differences in self-construals and social anxiety to be at least partially mediated by cultural group differences in threat appraisal.

### Study 1 method

#### Study 1 procedure

Two hundred and twelve native Japanese (116 females; *M* age = 20.88, *SD* = 2.23) and 249 European American (180 females; *M* age = 21.14, *SD* = 5.01) undergraduate students were recruited via subject pools from two large universities in Tokyo, Japan and Hawaii, U.S., respectively. To be included in the study, Japanese undergraduates needed endorsed Japanese ethnicity only, and wrote “Japanese” in response to the ethnicity and ethnic identity questions. Similarly, European American participants needed to be born in the United States and indicate their ethnicity as “White,” “Caucasian,” “European American,” or a specific European heritage (e.g., “German”). The two groups varied significantly in gender distribution^1^ (*χ^2^* [1] = 14.63; *p* < 0.001), but not in age or mothers’ and fathers’ years of education (*M* = 15.70, 15.55, *SD* = 1.78, 1.75, respectively, for Japanese; *M* = 15.46, 15.37, *SD* = 1.99, 2.32, respectively, for European Americans).

#### Study 1 measures

##### Social anxiety

We used the Social Interaction Anxiety Scale–6-item version (SIAS-6; Peters et al., 2011) to assess generalized forms of social anxiety symptoms present in daily interactions on a 5-point scale (0 = Not at all true of me, 4 = Extremely true of me); e.g., “I find myself worrying that I will not know what to say in social situations.” The SIAS-6 has shown strong evidence of validity, including a correlation of 0.88 with the original SIAS, diagnostic sensitivity, and sensitivity to changes in social anxiety during the course of psychotherapeutic treatment (Peters et al., 2011; Fergus et al., 2014). The Japanese translation of the original SIAS is widely used in Japan with comparable psychometric evidence (Kanai et al., 2004). More recently, Krieg and colleagues (Krieg et al., 2021) examined whether the SIAS-6 exhibited equivalent psychometric properties in Japanese and European Americans, by conducting a series of measurement invariance analyzes (Meredith, 1993; Little, 1997). Using multi-group confirmatory factor analyzes, they found that the two cultural groups responded to the measure in psychometrically similar ways, i.e., the SIAS-6 demonstrated the same single-factor structure, equivalent factor loadings, and comparable latent factor scores for Japanese and European Americans (i.e., scalar invariance; Little, 1997). The Cronbach’s alphas for the SIAS-6 were 0.74 for Japanese and 0.81 for European Americans in the current study.

##### Self-construal

The 30-item Singelis Self-Construal Scale (SCS; Singelis, 1994) was used to examine independent (15 items) and interdependent self-construals (15 items) on a 7-point Likert scale (1 = Strongly disagree, 7 = Strongly agree). Evidence for construct validity included higher levels of interdependent self-construal and lower levels of independent self-construal reported by Asian Americans in comparison to European Americans (Singelis, 1994). The translated Japanese measure has been widely used and validated among multiple native Japanese samples (e.g., Kleinknecht et al., 1997; Norasakkunkit and Uchida, 2011). In the current study, the Cronbach’s alphas for independent and interdependent self-construal were 0.78 and 0.75 for Japanese, and 0.85 and 0.78 for European Americans.

##### Threat appraisal

To assess threat bias as shown in biased appraisal of social threat, we conceptualized threat appraisal as the function of both anticipated consequence and likelihood of occurrence, i.e., those situations that meet both conditions of severe consequence and high likelihood would be perceived as a threat that likely leads to experience of social anxiety (Magnúsdóttir and Smári, 1999; Krieg and Xu, 2018). Thus, threat appraisal is related to not only evaluation of potential negative sequences of a social situation, but also how likely a social situation may occur.

To ensure that the social situations we used to measure threat appraisal are culturally relevant, we first conducted a pilot study (Krieg and Xu, 2018) using a situation sampling approach (Kitayama et al., 1997; Morling et al., 2002). The situation sampling approach typically involves (1.) asking members of a cultural group to freely generate a particular kind of situations (in our case, situations that may elicit social anxiety), and then (2.) redistributing (often randomly) these culturally relevant situations (or a subgroup of these situations) to a larger sample from the same cultural group. The situation sampling approach offers many methodological advantages, including the face validity of situations generated by and used with individuals of the same cultural backgrounds, as well as having the spread of situations randomly balanced across participants.

In the pilot study mentioned above, (Krieg and Xu, 2018) asked a group of 30 native Japanese and 30 European Americans to “create brief, specific situations where someone would feel socially anxious.” Although the length of the situation was not specified, situations raged between two and fifteen words, with a median of five words. Examples of the situations generated by Japanese included “asserting my opinion” and “not being recognized as a member of a group.” Examples of the situations generated by European Americans included, “accidentally taking someone else’s coffee” and “being told that what you are doing is wrong.” All of these situations were translated by two bilingual researchers either to or from Japanese in order to have linguistically equivalent translations of every situation (Brislin, 1970), resulting in a pool of 510 situations (313 by Japanese and 297 by European Americans; see Table 3 in Krieg, 2018 for a comprehensive list).

To ensure random redistribution of situations as emphasized in the situation sampling approach, Study 1 participants rated 15 randomly selected situations (from the situation pool mentioned above) generated by members of their own cultural group, as well as 15 randomly selected situations generated by members of the other culture group (30 situations total). For each situation, we asked participants to rate the extent to which they perceive the situation to hold a dire social consequence (i.e., “How bad would the consequences be?;” 1 = Not bad at all, 4 = Extremely bad) as well as the likelihood of its occurrence (i.e., “How likely is this situation going to occur?;” 1 = Not likely at all, 4 = Very likely). Following Magnúsdóttir and Smári (1999) and Smári et al. (2001), the product of these two scores (consequence rating ^X^ likelihood rating), averaged across 15 situations rated by each participant, was used as a marker of threat appraisal. The threat appraisal scores ranged from 2.51 to 6.76 for Japanese from 1.07 to 6.47 for European Americans. Given that each threat appraisal score was derived based on randomly selected different stimuli for each participant, internal consistency estimates could not be generated.

#### Study 1 analytic plan

**Figure 1:**
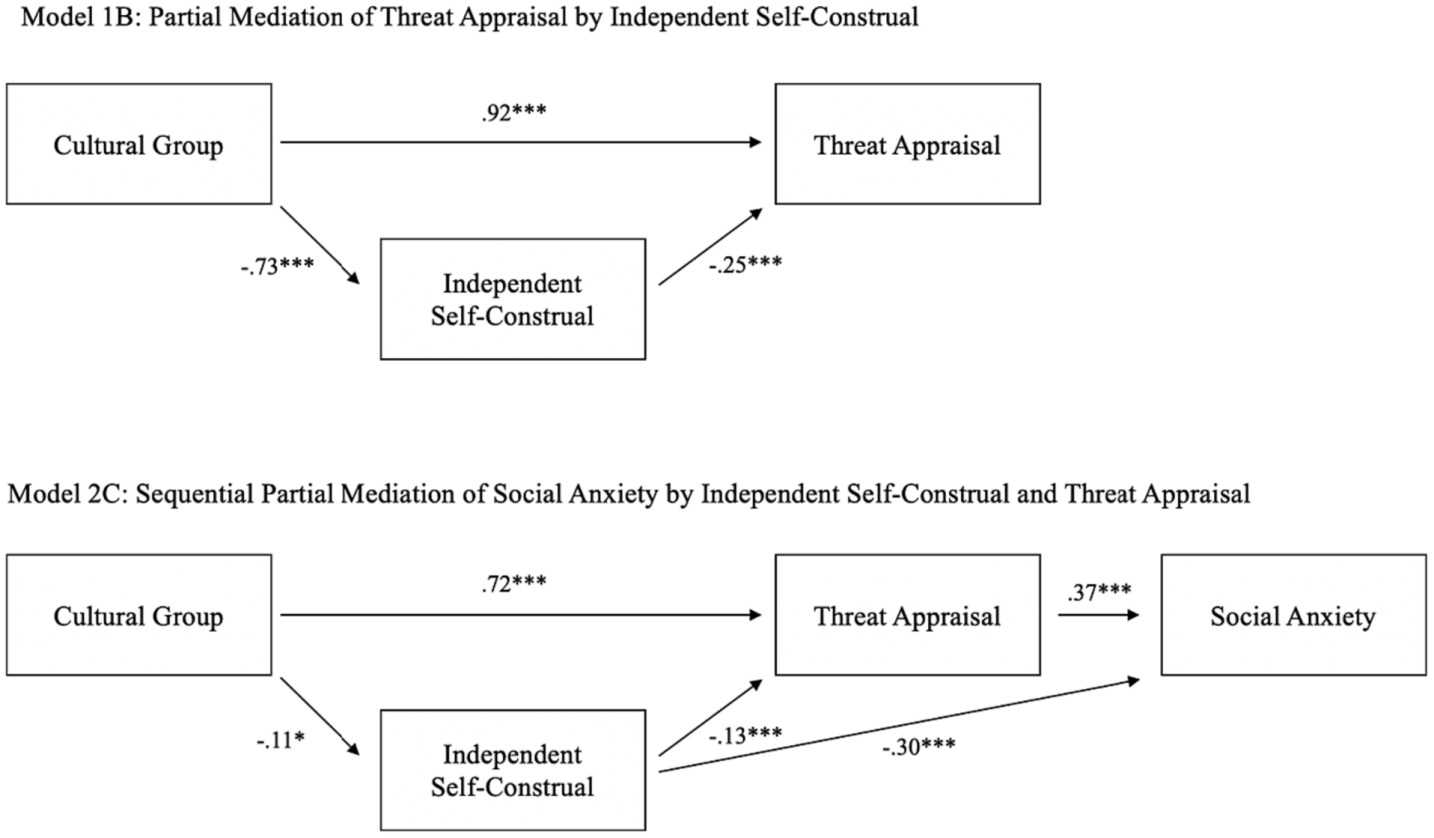
Model 2C in Study 1 (partial sequential mediation by IND and threat appraisal without direct effect of cultural group on social anxiety). Standardized coefficients were reported; for the cultural group variable, Japanese was coded as 1 and European Americans coded as 0; *p < 0.05; **p < 0.01; ***p < 0.001.

We first compared group means for each variable using ANCOVAs, partialialing out the variance associated with age and gender. Upon confirming higher social anxiety symptoms reported by Japanese than European Americans, we took a stepwise approach using structural equation modeling implemented through R package “lavaan” (Rosseel, 2012). Following Krieg and Xu (2018), we sequentially tested a series of models that gradually introduced self-construal and threat appraisal variables as the mediators to explain cultural group differences in social anxiety. Specifically, we first tested how higher interdependent and lower independent self-construals may help explain higher threat appraisal reported by Japanese than European Americans. We then added social anxiety as the outcome variable to the models to test our proposition that higher interdependent self-construal and lower independent self-construal may lead to higher threat appraisal which in turn results in higher social anxiety among Japanese than European Americans (see details below). To test these mediation models, we used a WLSMV estimator and z-scored all numeric variables in order to standardize their standard deviations. Given that the proposed mediations of self-construals and threat appraisal could occur with or without including the direct effect (e.g., the direct path from the cultural group variable to social anxiety; see Figure 1), we tested a series of competing models that varied in full (with the direct effect) or partial mediations (without the direct effect). To evaluate and compare model fit, we examined Comparative Fit Index (CFI), Tucker-Lewis fit index (TLI), Root Mean Square Error of Approximation (RMSEA), and Square Root Mean Residuals (SRMR). According to Hu and Bentler (1998, 1999) the recommended cutoff for the CFI and TLI is any value above 0.95. For the RMSEA and SRMR, the recombination is a value below the cutoff of 0.06 and 0.08, respectively.

### Study 1 results

Descriptive statistics as well as estimates of effect sizes in cultural group comparison were summarized in Table 1; Table 2 summarizes the correlation among all variables in Study 1.

**Table 1:** Means and standard deviations for all variables in Study 1 and Study 2.

Study 1	Japanese (*n* = 212)		European Americans (*n* = 249)		Cohen’s *d*
Variable	Mean	SD	Mean	SD	
Social anxiety	1.57	0.87	1.00	0.69	0.72
Independent SC	4.48	0.69	4.62	0.73	−0.20
Interdependent SC	4.48	0.65	4.52	0.65	0.05
Threat appraisal	4.68	0.70	2.90	0.96	2.11

Study 2	Japanese (*n* = 43)		European Americans (*n* = 47)		Cohen’s *d*
Variable	Mean	SD	Mean	SD	
Pre-ABMT					
Bias index	1.44	7.56	−4.19	8.02	0.57
Social anxiety	2.46	0.70	1.17	0.60	1.98
Independent SC	4.65	0.65	4.87	0.81	−0.15
Interdependent SC	4.64	0.63	4.72	0.84	−0.28

Variable	Mean	SD	Mean	SD	Cohen’s *d*
Post-ABMT					
*Attention toward threat condition (JN n = 22, EA n = 21)*					
Bias index	2.63	15.58	−0.04	10.43	0.20
Social anxiety	1.05	0.88	0.89	1.13	0.16
Change in GSR	1.29	0.54	1.33	0.39	0.04
BASA	2.25	0.45	1.80	0.39	1.06
*Attention away from threat condition (JN n = 21, EA n = 26)*					
Bias index	2.20	14.86	−3.92	10.22	0.48
Social anxiety	0.34	0.46	0.91	0.88	0.81
Change in GSR	1.07	0.28	1.06	0.17	0.04
BASA	2.03	0.48	1.99	0.35	0.10

JN, Japanese; EA, European American; SC, Self-Construal; GSR, Galvanic Skin Response; BASA, Behavioral Assessment of Speech Anxiety.

**Table 2:** Correlations among variables in Study 1.

	Japanese nationals (*n* = 212)			
Measure	1	2	3	4
1. Social anxiety	–	−0.29***	−0.03	0.19***
2. Independent SC	−0.45***	–	0.20***	−0.13*
3. Interdependent SC	0.10	0.23***	–	−0.02
4. Threat appraisal	0.29***	−0.26***	−0.00	–
	European Americans (*n* = 249)			

SC, Self-Construal; *p < 0.05; **p < 0.01; ***p < 0.001.

#### Cultural group differences in social anxiety, self-construals, and threat appraisal

Consistent with our first hypotheses, Japanese scored higher on social anxiety (*F* [1, 459] = 60.43; *p* < 0.001; Cohen’s *d* = 0.72) and Threat Appraisal (*F* [1,459] = 501.43; *p* < 0.001; Cohen’s *d* = 2.09), whereas European Americans scored higher on independent self-construal (*F* [1, 459] = 4.53; *p* = 0.03; Cohen’s *d* = −0.20). Unexpectedly, cultural group differences were not found for interdependent self-construal (*F* [1, 459] = 0.26; *p* = 0.61; Cohen’s *d* = 0.05).

#### Cultural group differences in independent self-construal and threat appraisal in relation to cultural group differences in social anxiety

##### Independent self-construal as the mediator in explaining cultural group differences in threat appraisal

Given the lack of cultural group differences in interdependent self-construal, we only examined how cultural group differences in independent self-construal may help explain (mediate) cultural differences in threat appraisal (see Figure 1). Table 3 shows comparisons of fit indices for the full and partial mediation models, with or without the direct effect of the cultural group variable (1 = Japanese, 0 = European American) on threat appraisal. As shown in Table 3, the full mediation Model 1A in Figure 1 did not fit the data satisfactorily: CFI = 0.55, TLI = 0.34, RMSEA = 0.66, SRMR = 0.35. The partial mediation Model 1B in Figure 1 is a just-identified model with zero degree of freedom. Therefore, we can only estimate path coefficients which, were all significant and thus retained in further model testing.

**Table 3:** Sequential mediation models for Study 1.

Model	Description	Study 1				
		DF	CFI	TLI	RMSEA	SRMR
Model 1A	Full mediation of IND on cultural group and threat appraisal	1	0.553	−0.342	0.661	0.352
Model 1B*	Just identified partial mediation of IND on cultural group and threat appraisal	0	NA	NA	NA	NA
Model 2A	Full sequential mediation by IND and threat appraisal	2	0.904	0.771	0.238	0.083
Model 2B	Partial sequential mediation by IND and threat appraisal with direct effect of cultural group on social anxiety	1	0.878	0.266	0.380	0.072
Model 2C*	Partial sequential mediation by IND and threat appraisal without direct effect of cultural group on social anxiety (Figure 1)	1	0.994	0.964	0.084	0.016
Model 2D	Just identified partial mediation model	0	NA	NA	NA	NA

IND, Independent Self-Construal; CFI, Comparative Fit Index; TLI, Tucker-Lewis Index; RMSEA, Root Mean Square Error of Approximation; SRMR, Standardized Mean Square Residual; *, Selected model.

##### Cultural group differences in independent self-construal and threat appraisal in relation to cultural group differences in social anxiety

Building on the partial mediation Model 1B, we added social anxiety as the outcome variable and tested four possible full and partial sequential mediations of independent self-construal and threat appraisal (see Table 3 for all fit indices). Model 2A, which tested a full double mediation by independent self-construal and threat appraisal, had poor model fit. Model 2B tested a partial double mediation by adding a path from the cultural group variable to social anxiety, but also had poor model fit. Model 2C tested a partial sequential mediation without the direct effect from the cultural group variable to social anxiety but including paths from independent self-construal and threat appraisal to social anxiety (see Figure 1). Model 2C fit the data satisfactorily; CFI = 1.00, TLI = 1.00, RMSEA = 0.02, SRMR = 0.01. Model 2D added a direct path from the cultural group variable to social anxiety to Model 2C making it a just-identified model but the path from cultural group to threat appraisal was not significant. Taken together, we selected Model 2C as our best-fitting model (see Table 3).

### Study 1 discussion

The results of Study 1 show a far more nuanced picture of the roles of self-construals and threat appraisal in explaining the cultural group differences in social anxiety. As expected, Japanese participants reported higher threat appraisal and social anxiety symptoms than European Americans. However, the two groups only differed in independent but not interdependent self-construal. A close examination of the literature indicates while earlier studies tended to demonstrate robust differences in interdependent self-construal between Japanese and European Americans (e.g., Ozawa et al., 1996; Sato and Cameron, 1999), more recent findings were more mixed (Bresnahan et al., 2005; Norasakkunkit et al., 2012), particularly when the Japanese samples were drawn from metropolitan areas. Some researchers speculate that the waning interdependent self-construal among Japanese found in some recent studies may be partly due to the continuing shift in orientation from traditional values to Western values among younger generations (Hamamura, 2017; Ogihara, 2017). Such value shift may also help explain the lack of group differences in interdependent self-construal between our European Americans and Japanese samples; the latter consisted of young college students from a metropolitan city in Japan.

The results of path analyzes provided preliminary support for the primary mediating pathway of our interest (dummy coded cultural group → cultural group differences in independent self-construal → cultural group differences in threat appraisal → cultural group differences in social anxiety). The findings are not only in line with the self-construal theory of social anxiety, but also offer insight into one possible mechanism that could help explain why lower independent self-construal may be related to higher social anxiety among Japanese than European Americans. Compared to European Americans, on average Japanese seem to downplay the importance of viewing oneself as a unique and autonomous entity in social settings, in order to avoid social conflict and maintain harmony with others. Such interpersonal tendencies may heighten their sensitivity to potential threat in social interactions, and potentially lead to more biased threat appraisal, as indicated by overestimation of both occurrence and negative consequences of social situations. Furthermore, much research has shown that overestimation of threat when evaluating social situations tend to precipitate and maintain social anxiety symptoms (e.g., MacLeod et al., 1986; Magnúsdóttir and Smári, 1999; Dalgleish et al., 2003; Heimberg et al., 2010; Singer et al., 2012). Thus, understanding the mediating role of threat appraisal represents an important first step in explaining the relations between cultural group differences in independent self-construal and social anxiety.

Similar to what was found in comparisons of Asian Americans to European Americans (e.g., Krieg and Xu, 2018), the mediating pathway mentioned above does not fully explain cultural group differences in social anxiety. Cultural group differences in social anxiety remained significant even after taking into account cultural group differences in independent self-construal and threat appraisal, suggesting that it is necessary to explore additional mechanisms that could further explain higher social anxiety reported by Japanese than European Americans. Study 2 was conducted to explore another facet of threat bias: selective attention to social threat in explaining cultural group differences in social anxiety.

## Study 2

While Study 1 focused on one aspect of threat bias that involves appraisal of social threat, Study 2 examined selective attentional bias, using a standardized dot-probe discrimination task (MacLeod et al., 1986; Amir et al., 2008, 2009) and the corresponding Attention Bias Modification Training (ABMT), in which participants were randomly assigned to Attend Threat and Attend Neutral conditions (see details below; MacLeod et al., 2002; Amir et al., 2011).

Specifically, Study 2 examined attentional bias and self-reported social anxiety among Japanese and European Americans both before and after ABMT. In addition, we also assessed behavioral (i.e., observations of anxious behavior in a speech task) and physiological (i.e., changes in skin conductance) markers of social anxiety after ABMT.

1. Before ABMT, similar to Study 1, we expected Japanese to report lower independent self-construal and higher social anxiety symptoms than European Americans. In addition, we expected Japanese, but not European Americans, to exhibit attentional bias toward threat (i.e., angry faces; see details below). Furthermore, similar to Study 1, we expected cultural group differences in pre-ABMT attentional bias to at least partially explain the relations between cultural differences in self-construals and pre-ABMT social anxiety.
2. Controlling for pre-ABMT attentional bias and social anxiety, after ABMT, we expected Japanese to continue to demonstrate stronger attentional bias than European Americans among those who were assigned to Attend Threat condition but expected diminished or nonsignificant cultural group differences in post-ABMT attentional bias among those who were assigned to Attend Neutral condition.
3. Controlling for pre-ABMT attentional bias and social anxiety, after ABMT, we expected Japanese to continue to report stronger social anxiety than European Americans among those who were assigned to Attend Threat condition but expected diminished or nonsignificant cultural group differences in post-ABMT social anxiety among those who were assigned to Attend Neutral condition.
4. Following Heeren et al. (2012a,b), we also included a speech task as a post-ABMT social stressor and examined the impact of ABMT on physiological (i.e., changes in skin conductance) and behavioral responses (i.e., observed anxious behavior) to this speech task. We expected Japanese and European Americans who were assigned to Attend Threat, but not those assigned to Attend Neutral condition, to differ in their physiological and behavioral response to the speech task as a social stressor.

### Study 2 method

#### Study 2 procedure

Forty-three Japanese (27 females; *M* age = 21.72, *SD* = 4.90) and 47 European American undergraduates (37 females^2^; *M* age = 21.83, *SD* = 1.88) were recruited from the same sites as in Study 1. Participants learned about the study in the form of an announcement in one of their psychology classes. They were given a choice between receiving extra credit in their class or $15.00 USD (¥1,500 JPY) upon completion of the experiment, which took approximately 45 min. Participants completed the experiment in a local psychology laboratory. Due to an unknown procedural error, one participant pre-ABMT attentional bias scores were not properly registered and subsequently removed from the dataset.

After signing the Informed Consent, participants were first administered the same self-construal scale and SIAS-6 as in Study 1, and the dot-probe discrimination task (see details below) before ABMT. Then, they were randomly assigned to Attend Threat (i.e., toward angry faces) or Attend Neutral (i.e., toward neural faces) ABMT condition. Participants completed the tasks on computers with exactly the same setup in the two university laboratories in the U.S. and Japan, with the researcher watching through a one-way mirror to unobtrusively monitor participant’s progress. Participants then completed the same dot-probe task and SIAS-6 to measure attentional bias and social anxiety after ABMT.

**Figure 2:**
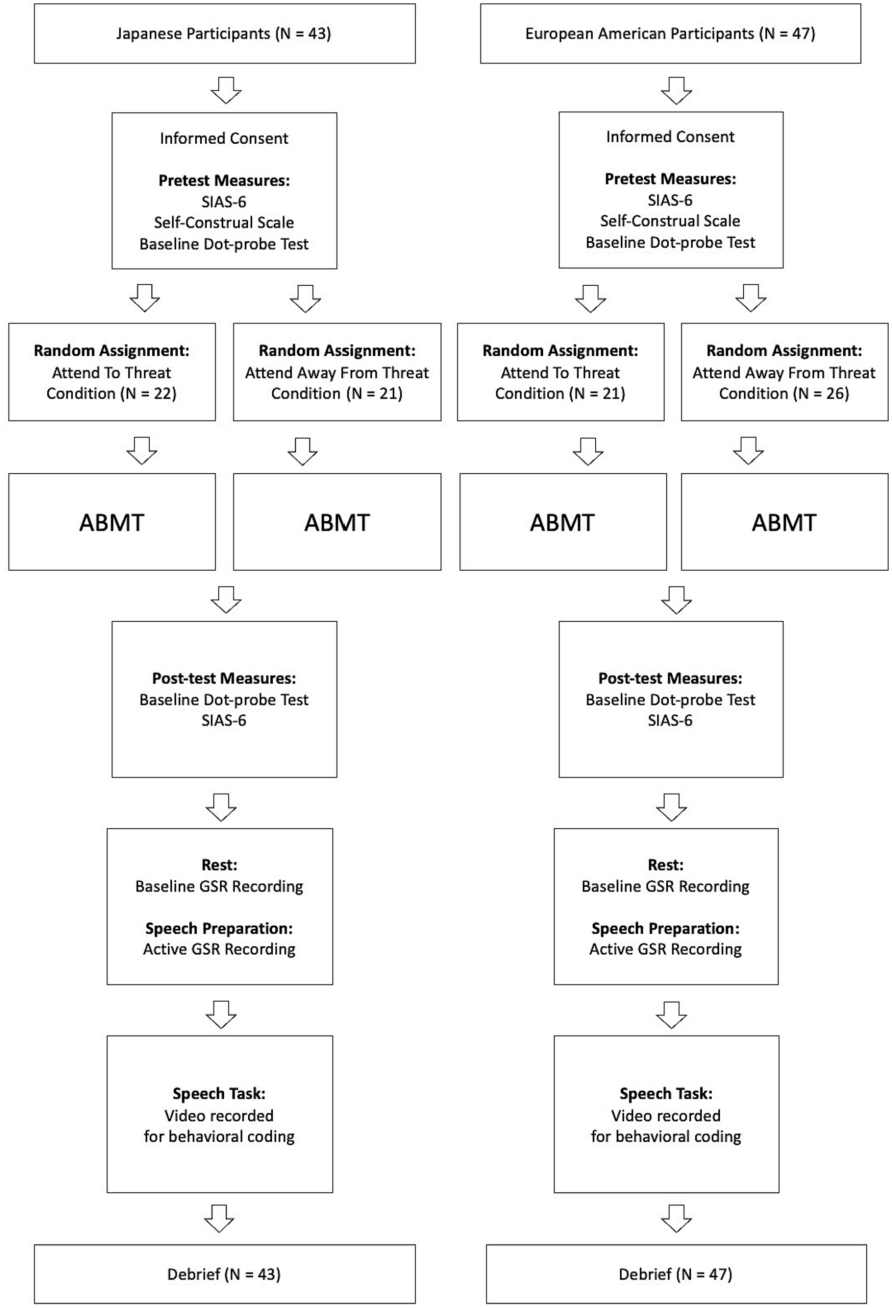
Flowchart of Study 2 procedures. SIAS-6 =; ABMT = Attention Bias Modification Training; GSR = Galvanic Skin Reactance.

Next, participants received instructions on a computer screen to rest quietly for one minute (resting phase). A countdown timer then appeared to alert participants how much time remained. After the one-minute timer expired, the on-screen instructions informed participants that they had been “selected for a speech task.” Following Heeren et al. (2012a,b), participants were instructed to “use the next two minutes to prepare a short speech about a negative emotional event that they had experienced in the past year” (preparation phase). A two-minute countdown timer then appeared on the screen. When the two minutes expired, the experimenter entered the room, asking participant to stand in a designated area in front of a video camera. The experimenter then left the room while participants’ speeches were video recorded (speech phase). The experimenter returned after participants signaled that they were done with their speeches, fully debriefed each participant, and asked them to sign an updated version of the informed consent that contained information about the impromptu speech task (see Figure 2 for a flowchart of Study 2 procedures). All participants signed the second informed consent and agreed to allow the data collected to be used in the current research.

We measured skin conductance (Galvanic Skin Response; GSR) during both the 1-min resting (baseline) and 2-min preparation phase (see details below) and focused on the GSR changes from the baseline to the preparation phase. We chose to assess GSR changes during preparation for speech (in comparison to the baseline) rather than during speech itself for both conceptual and methodological reasons (Edelberg, 1972). First, the peak anticipatory social anxiety would likely occur just before the event rather than during it. Second, because participant would be standing and recording the videos themselves, physical movement could disrupt the reading and add unwanted variance to the GSR measure. In addition to GSR, trained research assistants also coded videotaped speeches with regard to anxious behavior using a standardized protocol (see details below).

#### Study 2 Measures

##### Social anxiety before and after ABMT

We measured pre- and post-ABMT social anxiety using the same SIAS-6 (pre-ABMT Cronbach’s alphas: Japanese = 0.75, European American = 0.83; post-ABMT Cronbach’s alphas: Japanese = 0.77, European American = 0.82) as in Study 1.

##### Self-construal

The same Singelis Self-Construal Scale (SCS; Singelis, 1994) was used to assess independent self-construal (Cronbach’s alphas = 0.81 and 0.86 for Japanese and European Americans, respectively) and interdependent self-construal (Cronbach’s alphas = 0.71 and 0.75 for Japanese and European Americans, respectively).

##### Attentional bias before and after ABMT

To assess attentional bias before and after ABMT, participants completed a dot-probe discrimination task (MacLeod et al., 1986) modified from a similar task used in Heeren et al. (2012a,b) and Amir et al. (2011). The dot-probe task assesses preferential allocation of attention between two sets of stimuli displayed simultaneously on a screen. It is assumed that individuals are faster to respond to a probe that is presented in an attended, rather than unattended, region of a visual display (Posner et al., 1980). Thus, faster reaction time to the probe (e.g., lowercase letter “e” or “f” in the current study, see below) at the place of stimuli belonging to one set (e.g., angry faces) as compared to the other (e.g., neutral faces) demonstrates selective attention toward some defining feature of the stimuli set (e.g., direct social threat implied by angry faces).

We selected angry and neutral faces from a standardized set of European American and Japanese faces (Matsumoto and Ekman, 1989; four photos for each group, matched in various characteristics such as background, brightness, and size), so that the cultural backgrounds of participants would match those of the actor shown in face photos. Prior studies showed that angry faces indicate a non-ambiguous danger that requires immediate action and thus generally represent direct social threat for most cultural groups (Grillon and Charney, 2011; de Valk et al., 2015). This standardized set of face images had been widely used in previous cross-cultural studies of Japanese and European Americans with established evidence of reliability and validity (e.g., Matsumoto and Ekman, 1988, 1989).

In each trial, an angry face was randomly chosen to pair with a neutral face of the same actor; the two faces were randomly placed above and below center screen so that there is an approximately four-centimeter gap between the two. Each angry face was viewed two times, resulting in a total of 32 trials (16 angry faces, 8 per cultural group, paired with neutral faces of the same actor). The experiment repeated the set of 32 trials 3 times, meaning that each participant provided 96 responses. Each trial began with display of a black fixation cross in the center of a gray background in order to center participants’ gaze. After 500 milliseconds, the fixation cross disappeared and was replaced by two photographs, one angry face and one neutral face. Each pair of faces remained on the screen for 500 milliseconds before being replaced with either the lowercase letter “e” appearing in the top quadrant (where the upper picture was) or a lowercase letter “f” appearing in the bottom quadrant (where the lower picture was). Participants pressed the corresponding key to end the trial. Once the reaction time measured between letter stimulus onset and key press was recorded, that trial was saved and the next trial began. There was an inter-trial interval of 1,500 milliseconds.

Following a similar approach as Schmukle (2005) and Staugaard (2009), we operationalized attentional bias using the formula below:


$$\mathrm{attentional}\;\mathrm{bias}\;\mathrm{index}=\frac{1}{2}\left(\begin{array}{l} Mean_{\mathrm{reaction}\;\mathrm{time}\;for\;\mathrm{incongruent}\;\mathrm{trials}}\\ -\;Mean_{\mathrm{reaction}\;\mathrm{time}\;for\;\mathrm{congruent}\;\mathrm{trials}} \end{array}\right)$$

That is, attentional bias is defined as half of the mean difference in reaction time between incongruent trials (angry face and probe are in different locations) and congruent trials (angry face and probe are in the same location) and reflects which location the participant was attending to at the moment of response (Chen et al., 2002). Positive attentional bias index scores indicate attentional bias toward threat whereas negative attentional bias index scores suggest selective attention toward neutral stimuli, with a score of zero indicating a lack of attentional bias.

##### Attention Bias modification training (ABMT)

ABMT was based on the dot-probe discrimination task mentioned above (Amir et al., 2009; Amir and Conley, 2014), but was modified to direct attention either toward angry (toward threat) or neutral faces (away from threat). In the Attend Threat condition, the letter stimuli (either “e” or “f” randomly chosen) appeared in the place previously occupied by an angry face 80% (rather than 50% as used in the pre- and post-ABMT dot-probe tasks) of the time. This condition trained participants to expect the letter to appear where angry faces were positioned, and thus implicitly increased attention toward angry faces. Likewise, in the Attend Neutral condition, the letter stimuli appeared under the neutral face 80% of the time, training participants to look away from angry faces and toward neutral faces. Following Heeren et al. (2012a,b), participants in each training condition completed 560 trials delivered consecutively with 30 s breaks every 80 trials. According to a meta-analysis by Beard et al. (2012), 500 trials is sufficient to create a moderate temporary effect of ABMT in a single session. On average, the training took approximately 25 min.

As a manipulation check, we examined whether ABMT successfully changed the relative reaction times associated with the angry (threatening) versus neutral (non-threatening) faces pre- and post-ABMT, we applied a linear mixed-effects model with random intercepts, with participant, trial ID, and key preference (“e” or “f”) treated as covariates. In this model, we specifically tested a three-way face (angry or neutral) x training (toward or away from angry faces) x test (pre- versus post-) interaction effect to establish the effectiveness of the training. This interaction effect was significant (*B* = 11.72; *t* [1,11,178] = 2.26; *p* = 0.023), indicating that the training was effective in shifting participant’s attention to the targeted stimuli. Specifically, we found that participants in the Attend Threat condition displayed relatively faster reaction times to angry faces after ABMT compared to before (mean difference: −2.41 ms). Likewise, we found that participants in the Attend Neutral condition displayed relatively faster reaction times to neutral faces after ABMT compared to before (mean difference: −17.52 ms).

##### Galvanized skin reactivity (GSR) changes during the preparation relative to resting phase

Skin conductance, also known as galvanized skin reactivity (GSR), measures the ease of passage of electricity from one point to another across the surface of the epidermis. The conduction of electricity across this surface is facilitated by perspiration produced by eccrine sweat glands, which are especially sensitive to activity in the sympathetic nervous system via the electrodermal system (Edelberg, 1972; Hassett, 1978). We measured skin conductance using snap-connect Ag/AgCl electrodes placed on the volar surfaces of the medial phalanges of the right index and middle finger of each participant. The signal transmission between the electrode and the epidermis was facilitated via BIOPAC’s GEL101 isotonic skin conductance gel, described as having a 0.5% saline solution within a neutral base. A Shimmer 3 GSR unit (Shimmer Sensing, 2013, 2014) recorded the signal at 100 HZ with the measurement range set to 56 kOhms – 220 kOhms. The raw ADM output was calibrated via a linear equation using parameters specific to this measurement range, and returned as skin resistance measured in kOhms, which was then converted to skin conductance (measured in μSiemens) by multiplying each value by 0.001. We used the research guidelines by Dawson et al. (1990) to calibrate the parameters and conversions associated with the skin conductance measurement. Skin conductance values were first averaged for the 2-min preparation phase, and then divided by the average skin conductance value collected during the 1-min resting phase in order to take into account individual differences in baseline skin conductance.

##### Behavioral observations of speech anxiety

Anxious Behaviors exhibited during the speech task mentioned above were rated using the Behavioral Assessment of Speech Anxiety (BASA; Mulac and Sherman, 1974; Heeren et al., 2012b), a standardized behavioral assessment scale. The BASA assesses 18 specific behaviors, such as fidgeting, swallowing, and breathing heavy. Each behavior was coded on a 7-point Likert scale (1 = not at all, 7 = strong). To consider the possible impact of the cultural backgrounds of coders, five bilingual research assistants who varied in cultural backgrounds (two native Japanese, two Asian Americans, and one European American) who were blind to conditions assigned to participants, rated all the video recorded speeches using the BASA. The inter-rater reliability was calculated as an intra-class correlation of 0.91 (CI: 0.88–0.94). We first calculated the average ratings for each behavior across the five raters and then calculate the sum scores across all the 18 items (Cronbach’s alpha = 0.83) as the aggregated behavioral ratings of speech anxiety. The BASA has demonstrated evidence of internal consistency, inter-rater reliability, and concurrent validity with expert ratings of speech performance anxiety in prior studies (Mulac and Sherman, 1974; Heeren et al., 2011).

### Study 2 results

Descriptive statistics as well as estimates of effect sizes in cultural group comparison were summarized in Table 1, both before and after ABMT.

#### Cultural group differences in self-reported social anxiety and attentional bias before ABMT

Consistent with the first hypothesis, Japanese on average reported higher social anxiety than European Americans: (*F* [1, 88] = 88.36, *p* < 0.001; *Cohen’s d* = 1.98) before the ABMT. However, contrary to what was found in Study 1, Japanese and European Americans did not differ in independent (*F* [1, 88] = 0.25, *p* = 0.616; *Cohen’s d* = 0.11) and interdependent self-construals (*F* [1, 88] = 2.09, *p* = 0.152; *Cohen’s d* = 0.31).

Results on the cultural group differences in pre-ABMT attentional bias index were more complex than what was hypothesized. While European Americans demonstrated selective attentional toward *neutral* faces (as compared to 0 or “no attentional bias”): one-sample *t* [46] = −2.55, *p* = 0.01; *Cohen’s d* = −0.37, Japanese showed a lack of attentional bias toward either angry or neutral faces: one-sample *t* [41] = 1.23, *p* = 0.23; *Cohen’s d* = 0.19). Consequently, the two cultural groups differed in attentional bias scores: *F* [1, 87] = 7.18, *p* = 0.01; *Cohen’s d* = 0.57, not because Japanese exhibited more attentional bias toward threat as hypothesized, but because European Americans showed stronger selective attention toward neutral stimuli.

We then tested and compared two alternative mediation models (without the self-construal variables due to the lack of cultural group differences in both types of self-construals) in which pre-ABMT attentional bias either partially or fully mediated (i.e., with or without direct effect of dummy coded cultural group on social anxiety) the relation between dummy coded cultural group (predictor) and social anxiety (outcome). The full mediation model fit the data poorly (CFI = 0.16, TLI = −1.50, RMSEA = 0.79, SRMR = 0.21), whereas the partial mediation model was a just-identified model with zero degree of freedom. The mediating effect of attentional bias was only significant in the full mediation model: *B* = 0.13, *p* < 0.001, but became nonsignificant (*B* = 0.03, *p* > 0.05) when the direct effect of the cultural group on social anxiety was taken into account in the partial mediation model.

#### Cultural group differences in post-ABMT attentional Bias

To address our second hypothesis, we controlled for pre-ABMT attentional bias and social anxiety and conducted a 2 × 2 ANCOVA with post-ABMT attentional bias as the outcome variable, and cultural group and ABMT condition as the between-group factors. However, inconsistent with our hypothesis, none of the main and interaction effects were significant.

#### Cultural group differences in post-ABMT social anxiety

To address our third hypothesis, we controlled for pre-ABMT attentional bias and social anxiety and conducted a 2 × 2 ANCOVA with post-ABMT social anxiety as the outcome variable, and cultural group and ABMT condition as the between-group factors. There were significant main effects of cultural group (*F* [1, 83] = 5.84, *p* = 0.02; *Cohen’s d* = 0.51) and ABMT condition (*F* [1, 83] = 4.98, *p* = 0.03; *Cohen’s d* = 0.47), but these main effects were qualified by their interaction that was marginally significant: (*F* [1, 83] = 3.39, *p* = 0.07; *Cohen’s d* = 0.39). To better explain this interaction effect, we divided the sample by ABMT condition and examined cultural group differences. Consistent with our hypothesis, the cultural group difference in social anxiety was no longer statistically significant post-ABMT among those who were assigned to Attend Threat condition (*B* = 0.16, *p* = 0.61), whereas Japanese who were assigned to Attend Neutral condition, actually reported lower post-ABMT social anxiety than European Americans who were assigned to the same condition (*B* = −0.56, *p* = 0.01), reversing the trend observed in the extant literature (see Table 1 for details). The significant interaction between culture group and ABMT training condition both highlights the role of threat bias in explaining culture group differences in self-reported social anxiety and also suggests that participants in each group had a differential reaction to the training.

#### Cultural group differences in GSR changes when preparing for the speech task and anxious behavior during the speech task after the ABMT

To address our last hypothesis, we controlled for post-ABMT attentional bias and social anxiety, which were measured right before the assessment of GSR changes and anxious behavior and conducted 2 × 2 ANCOVAs with cultural group and ABMT condition as the between-subject factors, and GSR changes and anxious behavior as the outcome variables, respectively.

Inconsistent with our hypothesis, we only found a significant main effect of ABMT condition (*F* [1, 84] = 12.66, *p* < 0.001; *Cohen’s d* = 0.75) on the changes in GSR; regardless of cultural group, participants who were assigned to Attend Threat condition showed stronger increase in GSR from the baseline to the preparation phase for the speech task, than those who were assigned to Attend Neutral condition. There was no significant main effect of cultural group or interaction between cultural group and ABMT condition.

Consistent with our hypothesis, there was a significant interaction between cultural group and ABMT condition (*F* [1, 84] = 5.44, *p* = 0.02; *Cohen’s d* = 0.49) that qualified the main effect of cultural group differences in socially anxious behavior (*F* [1, 84] = 5.48, *p* = 0.02; *Cohen’s d* = 0.49). To better explain this interaction effect, we divided the sample by ABMT condition and examined cultural group differences. That is, Japanese only demonstrated more anxious behavior during the speech task than European Americans after they were exposed to Attend Threat condition (*B* = 0.45, *p* < 0.01), but the two cultural groups did not differ in anxious behavior after they experienced Attend Neutral condition (*B* = 0.04, *p* = 0.72). As with self-reported social anxiety, the statistically significant interaction effect demonstrated how experimentally manipulating threat bias impacts culture group differences in anxious behavior.

### Study 2 discussion

By applying the dot-probe discrimination task and the corresponding ABMT, Study 2 extended the examination of cultural group differences in threat bias to the process of selective attention. Overall, there were mixed support for our hypotheses. Similar to Study 1 and consistent with prior meta-analyzes, we were able to replicate higher social anxiety reported by Japanese than European Americans before ABMT. However, the two groups did not differ in either independent or interdependent self-construals, possibly due to the smaller sample size in Study 2. Likewise, Study 2 also consisted of a college sample from metropolitan areas, who likely had more exposure to Western values and media than other demographics in Japan (e.g., Hamamura, 2012; Ogihara, 2017). Prior studies, including Study 1, showed that AH-EH differences in self-construals were not always found (e.g., Norasakkunkit et al., 2012) and tend to be modest in effect size, and thus require relatively large samples to achieve desirable statistical power.

While there were cultural group differences in selective attention before ABMT, contrary to what we hypothesized, Japanese did not show significant attentional bias toward social threat, or angry faces. Rather, the cultural group differences in selective attention were largely accounted by selective attention toward neutral faces by European Americans. However, it was unclear whether this was due to preference of neutral faces or avoidance of angry faces among European Americans. Matsumoto and Ekman (1989) showed that when presented with the same angry expressions (which were used in the current study), Americans perceived them as more intense than Japanese. These cultural differences may lead to stronger avoidance of angry faces by European Americans. Alternatively, neutral faces may not be perceived as equally “neutral” among participants within the two cultural groups (e.g., Lee et al., 2008). Perhaps the perceptual differences between angry and neutral faces were more distinct for European Americans than Japanese, resulting in stronger selective attention among European Americans. Teasing apart these possibilities requires integrating additional conditions (e.g., pairs of happy versus neutral faces) in future studies.

Due to the lack of group differences in self-construals, we were not able to test a similar sequential mediation model as in Study 1. Although the mediating pathway “cultural group → selective attention → social anxiety” was significant when the direct effect of cultural group on social anxiety was not taken into account, the model fit was unsatisfactory. The fact that cultural group differences in social anxiety remained significant (i.e., the direct effect) in this mediation model suggests that cultural group differences in selective attention alone are not sufficient in explaining higher social anxiety among Japanese than European Americans. This was not surprising since Study 1 showed that at least the appraisal of threat also played an important mediating role. There is a need to integrate multiple mediating mechanisms in future studies with larger samples.

The post-ABMT findings were also mixed. While Japanese reported lower post-ABMT social anxiety than European Americans when they were assigned to Attend Neutral condition, they continued to report higher post-ABMT social anxiety when being assigned to Attend Threat condition. These results provide preliminary evidence on the possible causal role cultural differences in attentional bias may play in explaining higher social anxiety reported by Japanese than European Americans. However, this explanation was qualified by the lack of such “group ^x^ ABMT condition” interaction effects on post-ABMT attentional bias. When looking further into the group-level data on pre- and post-ABMT attentional bias index scores using one-sample t-tests, we found that ABMT seemed only effective in manipulating group-level changes in selective attention for European Americans: Japanese continued to show lack of selective attention as a group (albeit with a wide range of individual variations as shown in Table 1), whereas European Americans no longer showed attentional bias toward neutral faces after they had experienced Attend Threat condition, but continued to demonstrate selective attentional toward neutral faces among those who were assigned to Attend Neutral condition.

Does it mean that ABMT was not effective in changing group-level selective attention for Japanese? To explore this possibility, we turned our attention to the pre-ABMT attentional bias index scores. Despite the use of random assignment of ABMT conditions, the two Japanese groups seemed to have discrepant pre-ABMT scores with decent effect size (*Means* = 0.30 versus 5.45; *Cohen’s d* = −0.34), though the difference was not significant: *t* [40] = −1.11, *p* = 0.28, possibly due to large standard deviations (16.39 and 13.65) relative to the group means. Given the quasi-experimental nature of between-cultural group comparisons (i.e., cultural group membership cannot be manipulated), it appears more appropriate in future studies to match pre-ABMT attentional bias scores (which was unlikely in the current design), so that pre-ABMT attentional bias index scores can be made more comparable between the cultural groups. Furthermore, ABMT seemed effective in “changing” cultural group differences in social anxiety (which had much smaller standard deviations than the attentional bias scores). Thus, rather than claiming ABMT does not work for Japanese, it may be that individual variation in selective attention within Japanese and European groups can to some degree “mask” the between-group variation, requiring stronger manipulation to reveal the effect of ABMT, such as changing the manipulation trials from 80 to 100%, when directing attention to either angry or neutral faces (e.g., Pettit et al., 2020).

The cultural group differences in post-ABMT behavioral responses to a social stressor (speech task) were consistent with our hypotheses. As proposed by MacLeod et al. (2002) and others (e.g., Amir et al., 2008; Heeren et al., 2012a,b), manipulation of attentional response to threatening or neutral stimuli should predict later reactions to a subsequent stressful event such as giving a speech: directing attention toward threat could increase emotional vulnerability to the stressor whereas directing attention toward neutral stimuli could decrease emotional vulnerability. Thus, differential bias in attentional response to angry and neutral faces could result in a differential tendency to display anxious behavior in response to social stressors such as the speech task used in the current study.

The group comparison of post-ABMT GSR changes in preparation for the speech task failed to find the hypothesized “group ^x^ ABMT condition” interaction. Instead, for both Japanese and European Americans, being assigned to Attend Threat condition led to stronger GSR changes while they were preparing for the speech task. MacLeod et al. (2002) found that the manipulation of attentional bias had no direct effect on emotional responses either during or immediately after ABMT in the absence of a social stressor; that is, participants assigned to Attend Threat and Attend Neutral conditions did not differ in self-reported emotional states, even though they showed distinctive responses to a subsequent stressor task. Thus, it seems possible that unlike the speech task itself, simply preparing for a speech does not represent a strong social stressor since it may not activate self-consciousness and fear of negative evaluation that often accompany giving a speech in front of a video camera. It would be important to assess physiological changes during the speech task, perhaps by adapting an alternative physiological measure that is not as susceptible to movement and vocalization as GSR.

## General discussion

The current studies represent an initial effort of exploring roles of two different aspects of threat bias: threat appraisal and selective attention in cultural group differences in social anxiety between Japanese and European Americans. Our studies were guided by the cultural model of the self and attempted to test two pathways through which group differences in independent and interdependent self-construals may be manifested in culturally-tuned cognitive (Study 1) or attentional (Study 2) processing of social threat, which in turn result in higher social anxiety reported by Japanese than European Americans. Using both correlational (Study 1) and quasi-experimental designs (Study 2), and a wide range of methods, including self-reports, situation sampling, standardized computerized tasks, behavioral observations and physiological measures, we found mixed support for our hypotheses, particularly with regard to those on cultural group differences in selective attention.

Similar to Bresnahan et al. (2005) and Norasakkunkit et al. (2012), we only found lower independent self-construal among Japanese than European Americans in Study 1, but failed to reveal any group differences in either type of self-construal in Study 2. While the discrepancy in sample sizes likely played a role, between-cultural group differences in self-construals may not be as salient as originally thought and may often be “contaminated” by within-cultural individual variations as pointed out by Hamamura (2012) and Kitayama et al. (2006). The same problem also seemed to apply to between-cultural group differences (or lack thereof) in selective attention in Study 2. However, when selective attention was manipulated toward angry or neutral faces, its impact on post-ABMT cultural group differences in social anxiety and anxious behavior in response to a social stressor (speech task) became evident. Thus, it appears that detecting stronger between-group differences may require careful manipulation of independent or interdependent self-construals (e.g., Kühnen and Oyserman, 2002; Lin and Han, 2009) so the cultural group differences as well as the impact on various aspects of threat bias can be reliably evaluated.

The results of Study 1 and 2 also portrayed seemingly contradictory claims about the relations between cultural group differences in threat bias and social anxiety. The mediation models tested in Study 1 imply that the elevated threat appraisal among Japanese participants, as related to their lower independent self-construal, may be a reason why they reported higher social anxiety than European Americans. In contrast, Study 2 failed to find selective attentional bias toward threat among Japanese participants; instead, the findings suggest that selective attentional bias toward neutral faces among European Americans might be a reason why they reported lower social anxiety and exhibited less subsequent anxious behavior during the speech task than Japanese. This discrepancy might be, to some degree or less, related to a methodological issue that has plagued the literature on between-cultural differences: cultural group differences are relative. This means that a statistically significant between-group difference does not necessarily suggest whether it’s because of high score of one group, or low score of the other. In our case, the Likert scales for threat appraisal scores in Study 1 are arbitrary and provide no basis to argue for “high” biased appraisal among Japanese or “low” biased appraisal among European Americans. In contrast, we were able to compare attentional bias index scores in Study 2 to a referent zero, which indicates whether there was selective attention toward angry or neutral faces among either group.

In addition to the methodological issues, there is also a challenge to conceptualize and integrate Study 1 and 2’s results on biased appraisal and biased attention. Although our theoretical claims imply a correspondence between higher selective attention toward threat and more biased appraisal of threat among Japanese, possibly due to cultural differences in self-construals, our results failed to support this relation. Instead, we found both higher selective attention toward neutral stimuli and less biased appraisal threat among European Americans. In most cross-cultural literature, we tend to treat Western populations such as European Americans as the reference group, and the other comparative cultural group as “unique” and “unusual” and thus deviates from the Western “norm.” The findings of Study 2 may suggest another possibility: the well-replicated cultural group differences in social anxiety may to some degree indicate relatively low social anxiety reported by European Americans (rather than relatively higher social anxiety among Japanese); such cultural group differences may be partially accounted by European Americans’ preferential attention toward neutral stimuli (relative to threat) and relatively low appraisal of threat. Obviously, this speculation requires replication of the current findings and additional empirical tests in various domains of cultural group differences.

Other limiting methodological factors warrant cautious interpretation of the findings of current studies. Most significantly, an inevitable design limitation was having one cultural group tested in one location, and the other cultural group tested in another location. Despite our best efforts in keeping the laboratory conditions as similar as possible, there is a chance that at least some of the unique variance across cultural groups was attributable to differences in Study 2’s testing environment. This is especially worrisome given the elevated social anxiety in the Japanese sample in Study 2 compared to the Japanese sample in Study 1, whereas no such difference was found for European Americans. Similarly, Study 2’s sample size was modest, and the data generated by an undergraduate sample may not be readily generalizable to other populations, especially with the noted generational differences in self-construal.

Despite its theoretical appeal and popularity, our findings showed that the self-construal theory might not be sufficient in explaining AH-EH differences in threat bias and social anxiety, particularly attentional bias. This limitation may be partly due to waning cultural group differences in interdependent and independent self-construals because of globalization, but it also points to a need for alternative theoretical frameworks that could capture cultural differences in relatively effortless and to a large extent unconscious processes influencing selective attention. For instance, it may be beneficial to examine the contexts within which certain expressions are naturally presented for each cultural group. If members of one cultural group have a learning history of neutral faces being paired with negative social reinforcement (e.g., the withdrawal of a smile) or positive social punishment (e.g., verbal reprimand), it may still automatically evoke a sense of impending social threat in a similar way that an angry face is hypothesized. Supporting this speculation, prior research has found that “neutral faces” are not always perceived by all participants as neutral (Lee et al., 2008) and that this perception is influenced by contextual information (Suess et al., 2013) and learning history (Albohn and Adams, 2021). Krieg (2020) recommended a contextual-behavioral model for understanding cultural differences in social anxiety, specifically focused on antecedent conditions and social rewards and consequences for socially anxious behavior. Perhaps this model can be extended to include group differences in learning history to better account for differences in automatic associations between specific facial expressions and social threat for groups of Japanese and European Americans.

## Data availability statement

The raw data supporting the conclusions of this article will be made available by the authors, without undue reservation.

## Ethics statement

The studies involving humans were approved by University of Hawaii at Manoa, Office of Research Compliance, Human Studies Program (CHS #22377). The studies were conducted in accordance with the local legislation and institutional requirements. The participants provided their written informed consent to participate in this study.

## Author contributions

AK and YX: study conception and design, analysis and interpretation of results. AK: data collection and draft manuscript preparation. YX: manuscript editing and feedback. All authors contributed to the article and approved the submitted version.

## Funding

This study was funded in part by the U.S. Department of Education’s Fulbright-Hays Dissertation Development Award (2015-2016), the Japan-America Society of Hawaii’s Crown Prince Akihito Scholarship (2016-2018), and the Japanese Society for the Promotion of Science’s Grants-in-Aid (2021-2024).

## Conflict of interest

The authors declare that the research was conducted in the absence of any commercial or financial relationships that could be construed as a potential conflict of interest.

## Publisher’s note

All claims expressed in this article are solely those of the authors and do not necessarily represent those of their affiliated organizations, or those of the publisher, the editors and the reviewers. Any product that may be evaluated in this article, or claim that may be made by its manufacturer, is not guaranteed or endorsed by the publisher.

## Footnotes

^1^Due to the imbalanced gender composition between Japanese and European American samples, we repeated the group comparison analyses with both gender and age as covariates. All cultural group differences were replicated, and neither the gender or age covariates were statistically significant on any variable. Because of this, neither gender or age was not modeled in the subsequent mediation models.

^2^Due to the difference in gender composition of two samples, we repeated analysis with and without gender and age as covariates. However, the primary findings were replicated regardless of whether gender or age were included as covariates. Similarly, neither gender or age had a statistically significant relationship with independent or interdependent self-construal.
